# Using C-doped TiO_2_ Nanoparticles as a Novel Sonosensitizer for Cancer Treatment

**DOI:** 10.3390/antiox9090880

**Published:** 2020-09-17

**Authors:** Chun-Chen Yang, Chong-Xuan Wang, Che-Yung Kuan, Chih-Ying Chi, Ching-Yun Chen, Yu-Ying Lin, Gin-Shin Chen, Chun-Han Hou, Feng-Huei Lin

**Affiliations:** 1 Department of Materials Science and Engineering, National Taiwan University, Taipei 10617, Taiwan; d03527007@ntu.edu.tw (C.-C.Y.); r05527013@ntu.edu.tw (C.-X.W.); 2 PhD Program in Tissue Engineering and Regenerative Medicine, National Chung Hsing University, Taichung 40227, Taiwan; 090115@nhri.edu.tw (C.-Y.K.); d103001707@mail.nchu.edu.tw (C.-Y.C.); 024016@nhri.edu.tw (Y.-Y.L.); 3 Institute of Biomedical Engineering and Nanomedicine, National Health Research Institutes, Miaoli County 35053, Taiwan; chingyun523@nhri.edu.tw (C.-Y.C.); gschen@nhri.edu.tw (G.-S.C.); 4 Department of Biomedical Sciences & Engineering, National Central University, Taoyuan City 32001, Taiwan; 5 Department of Orthopedic Surgery, National Taiwan University, Taipei 10617, Taiwan; 6 Institute of Biomedical Engineering, National Taiwan University, Taipei 10617, Taiwan

**Keywords:** sonodynamic therapy, carbon doped titanium dioxide, sonosensitizers, ultrasound, cancer treatment, breast cancer treatment

## Abstract

Sonodynamic therapy is an effective treatment for eliminating tumor cells by irradiating sonosentitizer in a patient’s body with higher penetration ultrasound and inducing the free radicals. Titanium dioxide has attracted the most attention due to its properties among many nanosensitizers. Hence, in this study, carbon doped titanium dioxide, one of inorganic materials, is applied to avoid the foregoing, and furthermore, carbon doped titanium dioxide is used to generate ROS under ultrasound irradiation to eliminate tumor cells. Spherical carbon doped titanium dioxide nanoparticles are synthesized by the sol-gel process. The forming of C-Ti-O bond may also induce defects in lattice which would be beneficial for the phenomenon of sonoluminescence to improve the effectiveness of sonodynamic therapy. By dint of DCFDA, WST-1, LDH and the Live/Dead test, carbon doped titanium dioxide nanoparticles are shown to be a biocompatible material which may induce ROS radicals to suppress the proliferation of 4T1 breast cancer cells under ultrasound treatment. From in vivo study, carbon doped titanium dioxide nanoparticles activated by ultrasound may inhibit the growth of the 4T1 tumor, and it showed a significant difference between sonodynamic therapy (SDT) and the other groups on the seventh day of the treatment.

## 1. Introduction

Cancer has been the leading cause of death in the US for 40 consecutive years. In 2019, there were 606,880 deaths from cancer projected to occur in the US [1]. Surgery, radiotherapy and chemotherapy are the most fundamental and effective cancer treatments. Nevertheless, it is hard to remove tumor cells comprehensively via surgery; radiotherapy and chemotherapy may not only kill the cancer cells but cause harm to healthy cells nearby and make patients feel fatigue. Immunotherapy is likely to interfere with the immune system and cause autoimmune disease. Hyperthermia is probably resisted by cancer cells after several heat treatments. Photodynamic therapy (PDT) is limited due to the shallow penetration depth of light sources into tumor tissue. In previous studies, we used X-ray as an alternative light source which provided a novel therapeutic approach for deep-seated tumor/cancer treatment [2,3,4]; however, the annual radiation dose limit was another issue. Thus, an alternative therapy with fewer side effects was proposed by Umemura and Yumita, called “Sonodynamic therapy (SDT)” [5]. SDT can focus the ultrasound energy on the deeply located tumor site, which overcomes the shortcoming of PDT. SDT is considered to be a safer and more acceptable therapy for patients compared to radiotherapy and chemotherapy [6]. It is noninvasive, and the apparatus is relatively inexpensive [7,8].

SDT consists of three basic elements: ultrasound, sonosensitizer and oxygen molecules. The mechanism of SDT is that the nonthermal effect of acoustic cavitation generated by sonoluminescence, and the sonoluminescent light activates the sonosensitizer, leading to the electronic excitement of the sonosensitizer [7]. When the excited sonosensitizer decays back to the ground state, the released energy transfers to oxygen to generate the highly reactive singlet oxygen (^1^O_2_) [9]. Meanwhile, the energy may lead to pyrolysis reaction of the water near the exposed site of ultrasound and generate hydroxyl radicals (•OH). These reactive oxygen species (ROS) may cause the death of the tumor cells afterwards [10]. ROS plays an important role in cellular signaling pathways, such as metabolism, growth, differentiation and death signaling, and react with molecules by reversible oxidative modifications. Excess generation of ROS may cause cell senescence and death to intracellular biomacromolecules, such as protein, lipid, RNA and DNA, via oxidative damage [11].

Ultrasound is a mechanical wave with periodic vibrations in a continuous medium at frequencies greater than 20 kHz [12]. Ultrasound is able to penetrate tissue with less attenuation of energy. Therefore, it can be applied to medical diagnosis and therapeutic use. For a medical diagnosis purpose, the ultrasound is irradiated at a frequency of 2.0 to 28.0 MHz with low-energy irradiation to prevent tissue from damaging. For therapeutic use, the ultrasound is irradiated at a frequency of 0.5 to 3.0 MHz with higher doses of energy to generate the desired biological results [13]. For SDT, low-intensity ultrasound is used to induce the non-thermal and sono-chemical effects to activate sonosensitizer to cause the damage and even the death of tumor cells [14]. The non-thermal effect of ultrasound in SDT is cavitation that involves formation, growth and collapse of cavitation bubbles [7]. Under ultrasound irradiation, the static pressure of the aqueous solution decreases below the vapor pressure, and water may evaporate into gas bubbles. The cavitation bubbles nucleate in the presence of impurities or pre-existing bubbles in solution and oscillate in the phase under irradiation [15]. During the ultrasound irradiation, bubbles grow increasingly larger and stop growing when the static pressure equals the vapor pressure. They may start to break down from its weakest spot when the static pressure exceeds the vapor pressure, and then collapse (known as inertial cavitation) led to a highly concentrated energy release [16,17]. The released energy leads to the pyrolysis reaction of the water, which generate (•OH) and short light pulses (known as sonoluminescence) [18]. Sonoluminescence involves intense ultraviolet-visible light, which can excite sonosensitizer to generate ROS [19].

Sonosensitizers play a critical role in SDT that can enhance the effect of ultrasound. The development of sonosensitizers had grown swiftly in recent decades due to the known mechanisms of cell apoptosis for SDT [20]. The porphyrin-based sonosensitizers, such as photofrin, hematoporphyrin, 5-ALA (5-aminolevulinic acid) and chlorin-e6, are the most often used sonosensitizers in SDT research [7]. However, porphyrin-based sonosensitizers have phototoxicity on the skin that may affect both tumor cells and normal cells under a certain wavelength of light or energy irradiation in PDT studies, which means that this issue may also take place in SDT [20]. On the other hand, most sonosensitizers were hydrophobic and easy to aggregate in physiological condition, leading to a reduction in their ROS production [21]. Nonetheless, the development of nanoparticles shows a promising potential to solve these problems. Among many nanosensitizers, titanium dioxide (TiO_2_) has attracted the most attention due to its properties [21]. TiO_2_ is widely used in many territories based on low toxicity, high stability, high photocatalytic activity and low cost [22,23]. Compared to porphyrin-based sonosensitizers which are quickly degraded under oxidizing conditions, TiO_2_ exhibits high stability because it is highly resistant to degradation by ROS. TiO_2_ exhibits three kinds of crystal structures, namely anatase, rutile and brookite. Anatase and rutile are the most common in the utilization of crystal structures, and brookite is less used in industrial application. Even though anatase (E_g_ = 3.2 eV) has a wider bandgap than rutile (E_g_ = 3.0 eV), anatase shows higher photoactivity due to its larger specific surface area that anatase is more suitable to be used as a photocatalyst [24]. In previous studies, the anatase structure of TiO_2_ has been utilized as a sonocatalyst to generate ROS under ultrasound irradiation [10,14]. Nonetheless, the wide bandgap of anatase requires a greater energy to trigger. Carbon has previously been doped in the semiconductors to form a new valence band, thus narrowing the bandgap [25]. The addition of carbon may give TiO_2_ an excess of conducting electrons or holes which is important for lowering the bandgap [23,24].

Hence, in this study, the sonosensitizer C-doped TiO_2_ was synthesized through doping carbon into the anatase structure of TiO_2_ to diminish the bandgap. A square wave of the ultrasound at a resonant frequency of 1.0 MHz, intensity of 0.33 MPa and duty cycle of 50% was used to induce the inertial cavitation and generate sonoluminescent light to activate the synthesized C-doped TiO_2_.

## 2. Materials and Methods

### 2.1. Preparation of C-Doped TiO_2_

C-doped TiO_2_ was synthesized by the sol-gel method [23]. First, 2 g of Pluronic^®^ F127 (Sigma-Aldrich, St. Louis, MO, USA) was dissolved in 40 mL 95% ethanol completely with vigorous stirring. Then, 5 mL titanium(IV) isopropoxide (TIP, Ti(OCH(CH_3_)_2_)_4_, purity > 97%, Sigma-Aldrich) was added into the solution with magnetic stirring of 600 rpm. An amount of 3 g of D-(+)-glucose (C_6_H_12_O_6_, Sigma-Aldrich), which was used as the carbon source, was dissolved in 6 mL ddH_2_O. Then, the glucose solution was dropped into the TIP/Pluronic^®^ F127 solution. The mixed solution was kept vigorously stirring at room temperature for 30 min. The precipitate was collected by centrifugation at 5000 rpm and washed with 95% ethanol for three times. The precipitate was then calcined at 400 °C for 2 h to obtain particles.

### 2.2. Material Characterization

The crystal structure of the synthesized C-doped TiO_2_ was determined by X-ray diffraction (XRD; Rigaku TTRAX 3) with Cu Kα radiation at a speed of 2° per minute at 40 kV and 30 mA from 20° to 60°.The surface morphology and particle size of C-doped TiO_2_ were characterized by a scanning electron microscope (SEM; Nova Nano SEM 450). The structure and diffraction pattern of the material were analyzed by a transmission electron microscope (TEM; JEOL 2010F). The particle size of the material was reconfirmed via Zetasizer (Malvern Nano ZS). The chemical composition of the material was analyzed by an energy dispersive spectrometer (EDS; Nova Nano SEM 450) SEM attachment. To confirm that carbon had been doped in titanium dioxide and formed the bonding, C-doped TiO_2_ was measured by auger electron spectroscopy (AES; JEOL JAMP 9510F) and X-ray photoelectron spectroscopy (XPS; Theta Probe).

### 2.3. Ultrasound Apparatus

**Figure 1:**
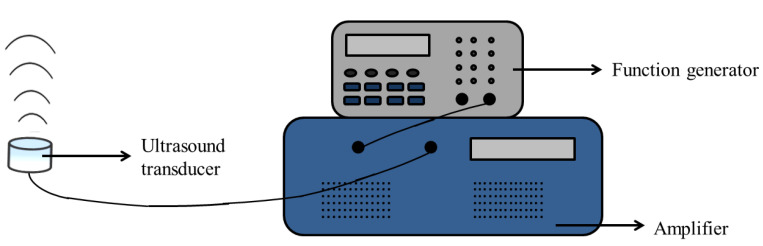
Ultrasound apparatus. The ultrasound transducer with a diameter of 2.0 cm was fixed with aluminum block by a rubber band to keep the transducer facing upward in the degassed water tank.

Ultrasound irradiation was conducted with a function generator (Agilent 33521A) at a resonant frequency of 1.0 MHz and a duty cycle of 50% and amplified by a power amplifier (E&I 1040L) to generate a square wave with a negative pressure of 0.33 MPa and intensity of 1.8 W/cm^2^ for 90 s in the whole experiment. Figure 1 shows the entire apparatus.

### 2.4. Preparation of Terephthalic Acid Solution and Evaluation of Ultrasound Parameter

First, 2 mM terephthalic acid (TA; Sigma-Aldrich) was dissolved in 800 mL ddH_2_O. During ultrasound treatment, the experiments should be carried out under alkaline condition. Hence, 5 mL 1 M of NaOH was added in the solution. TA solution was stirred for 1 h under 4 °C and dark conditions to avoid photochemical reaction [26].

The experiment was divided into two groups. No US group was named as the control group: the US group was the group irradiated by ultrasound. The fluorescence signal intensity was measured by a multi-label plate reader with excitation and emission wavelengths at 310 and 420 nm, respectively.

### 2.5. Cell Culture and Animal Model

Briefly, L929 cells obtained from National Health Research Institutes were cultured in MEM medium (Minimum Essential Media, Sigma-Aldrich) with 10% Fetal bovine serum (FBS, Gibco) and 1% Antibiotic-Antimycotic (Gibco) and maintained in a 5% CO_2_ atmosphere at 37 °C.

Breast cancer 4T1 cells obtained from National Health Research Institutes were used as model cancer cells in this study. The 4T1 cells were cultured in RPMI medium (RPMI-1640 Media (Sigma-Aldrich) with 10% Fetal bovine serum (FBS, Gibco) and 1% Antibiotic-Antimycotic (Gibco) and maintained in 5% CO_2_ atmosphere at 37 °C.

6-week-old male BALB/c nude mice were used as a xenograft animal model in this study. The nude mice were injected subcutaneously with 2 × 10^6^ 4T1 cells suspended in 200 μL 1X PBS into the right thigh. All the animal experiments were approved by National Health Research Institutes (NHRI-IACUC-107013).

### 2.6. Evaluation of Biocompatibility for the Synthesized C-Doped TiO_2_

The evaluation of biocompatibility in this study was based on International Standard ISO 10993. The cell viability induced by the synthesized C-doped TiO_2_ was determined by the WST-1 test (TAKARA). L929 cells were seeded on a 96-well cell culture plate with a cell density of 10^4^ cells per well and incubated in 5% CO_2_ atmosphere at 37 °C. Briefly, 0.2 g/mL of the materials was immersed in MEM medium for 16 to 24 h in 5% CO_2_ atmosphere at 37 °C as material extracts. The control group included L929 cells without treatment. Zinc diethyldithiocarbamate (ZDEC, Sigma-Aldrich) and aluminum oxide (Sigma-Aldrich) were indicated as the “Positive Control” and “Negative Control” group, respectively. The C-doped TiO_2_ group included cells cultured with C-doped TiO_2_. After one-day incubation, the previous solution in each well was removed, and 100 μL of the supernatant of the material extracts was added into each well. After one-day incubation, the previous solution in each well was removed, and 100 μL fresh MEM medium with 10% WST-1 reagent was added. After 2 h incubation in 5% CO_2_ atmosphere at 37 °C kept in the dark, the solution in each well was collected to measure absorbance at 490 nm by a microplate reader.

### 2.7. Detection of Cellular ROS Generation

ROS generation induced by the synthesized C-doped TiO_2_ in SDT was measured by a DCFDA-cellular ROS detection assay kit (abcam). 4T1 breast cancer cells were seeded on 12-well cell culture plates with a cell density of 6 × 10^4^ cells per well and incubated in 5% CO_2_ atmosphere at 37 °C. After one-day incubation, the previous solution in each well was removed, and 1 mL 15 mg/mL synthesized C-doped TiO_2_ in RPMI medium was added into each well. After 6 h incubation, ultrasound was then irradiated from the bottom of the cell culture plates in degassed water. The distance between ultrasound transducer and the bottom of the cell culture plate was around 5 mm. After US treatment, the cell culture plates were incubated for 2 h. The previous solution in each well was then removed and washed once with 1× PBS. An amount of 1 mL 20 μM 2′,7′ –dichlorofluorescin diacetate (DCFDA, abcam) in 1× buffer was added into each well for 30 min in 5% CO_2_ atmosphere at 37 °C in the dark. The previous solution in each well was removed, and cells were washed with 1× PBS once. An amount of 1 mL 1× PBS was added into each well. The fluorescence signal intensity was measured by a multilabel plate reader with excitation and emission wavelengths at 485 and 535 nm, respectively [27]. The control group included 4T1 cells without treatment. The C-doped TiO_2_ group included cells cultured with C-doped TiO_2_. The US group included cells subjected to ultrasound irradiation. The SDT group included cells cultured with C-doped TiO_2_ and subjected to ultrasound irradiation.

### 2.8. Evaluation of the Synthesized C-Doped TiO_2_ in SDT In Vitro

WST-1 and LDH tests were used to evaluate the cell viability and cytotoxicity of the synthesized C-doped TiO_2_ in SDT. In vitro, 6 × 10^4^ 4T1 cells suspended in 1 mL RPMI medium were seeded on 12-well cell culture plates and incubated in 5% CO_2_ atmosphere at 37 °C. After one-day incubation, the previous solution in each well was removed, and 1 mL 15 mg/mL synthesized C-doped TiO_2_ in RPMI medium was added into each well. After 6 h incubation, ultrasound was then irradiated from the bottom of the cell culture plates in degassed water. The distance between the ultrasound transducer and the bottom of the cell culture plate was around 5 mm. After ultrasound treatment, the cell culture plates were incubated for one day. For the WST-1 test, the previous solution in each well was removed, and 400 μL fresh RPMI medium with 10% WST-1 reagent was added. After 1 h incubation in 5% CO_2_ atmosphere at 37 °C kept in the dark, the solution in each well was collected to measure absorbance at 490 nm by an Elisa reader. The control group included 4T1 cells without treatment. Zinc diethyldithiocarbamate (ZDEC) and aluminum oxide were indicated as the “Positive Control” and “Negative Control” group, respectively. The C-doped TiO_2_ group included cells cultured with C-doped TiO_2_. The US group included cells subjected to ultrasound irradiation. The SDT group included cells cultured with C-doped TiO_2_ and subjected to ultrasound irradiation.

For the LDH test, 50 μL lysis solution was then added into each well of the Lysis group and then incubated in 5% CO_2_ atmosphere at 37 °C for 30 min. Fifty μL of supernatant from each well was then transferred to a 96-well cell culture plate, and 50 μL LDH reagent was added into each well. After 15 min of reaction at room temperature in the dark, the cytotoxicity was measured by a microplate reader with absorbance at 490 nm. The C-doped TiO_2_ group included cells cultured with C-doped TiO_2_. The US group included cells subjected to ultrasound irradiation. The SDT group included cells cultured with C-doped TiO_2_ and subjected to ultrasound irradiation.

### 2.9. Evaluation of SDT in In Vivo Tumor Growth

There were 4 different treatments exerted on nude mice: (1) the control group was injected with 200 μL 1× PBS at day 0 and day 7 without ultrasound irradiation; (2) the C-doped TiO_2_ group was injected with 200 μL synthesized C-doped TiO_2_ (150 mg/mL) in 1× PBS at day 0 and day 7 without ultrasound irradiation; (3) the US group was injected with 200 μL of 1× PBS at day 0 and day 7 with US irradiation; (4) the SDT group was injected with 200 μL synthesized C-doped TiO_2_ (150 mg/mL) in 1× PBS at day 0 and day 7 with US irradiation. There were 5 nude mice in each group.

The tumor volume was measured by an electronic slide caliper using Equation (1), where L is the longest dimension and W is the shortest dimension, parallel to the mouse body [28].

V = 0.5 × L × W^2^ (1)

### 2.10. Histopathology

During sacrifice, cardiac puncture was used to obtain the blood samples. The samples were collected into tubes with 7.5% EDTA solution. Tumor and internal organs, including heart, liver, spleen, lung and kidney were also harvested and then fixed with 4% formaldehyde. These organs were embedded into the paraffin and further stained with hematoxylin and eosin (H&E) for histologic examination.

### 2.11. Statistics

All data are indicated as the mean ± standard deviation. Statistical analysis was performed using one-way ANOVA followed by multiple comparisons with the Dunnett test, and the difference between the groups was deemed to be statistically significant when *p* < 0.05.

## 3. Results

**Figure 2:**
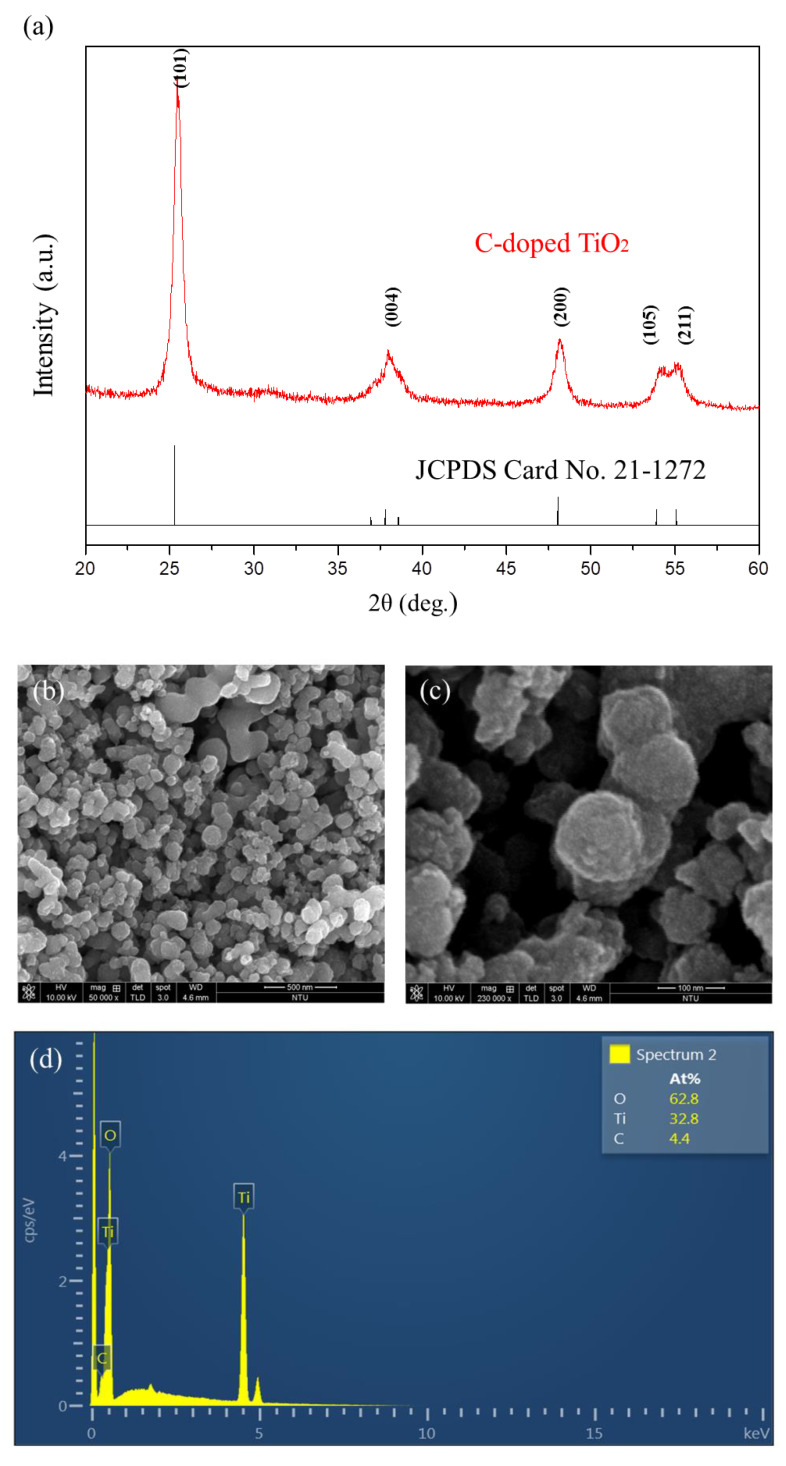

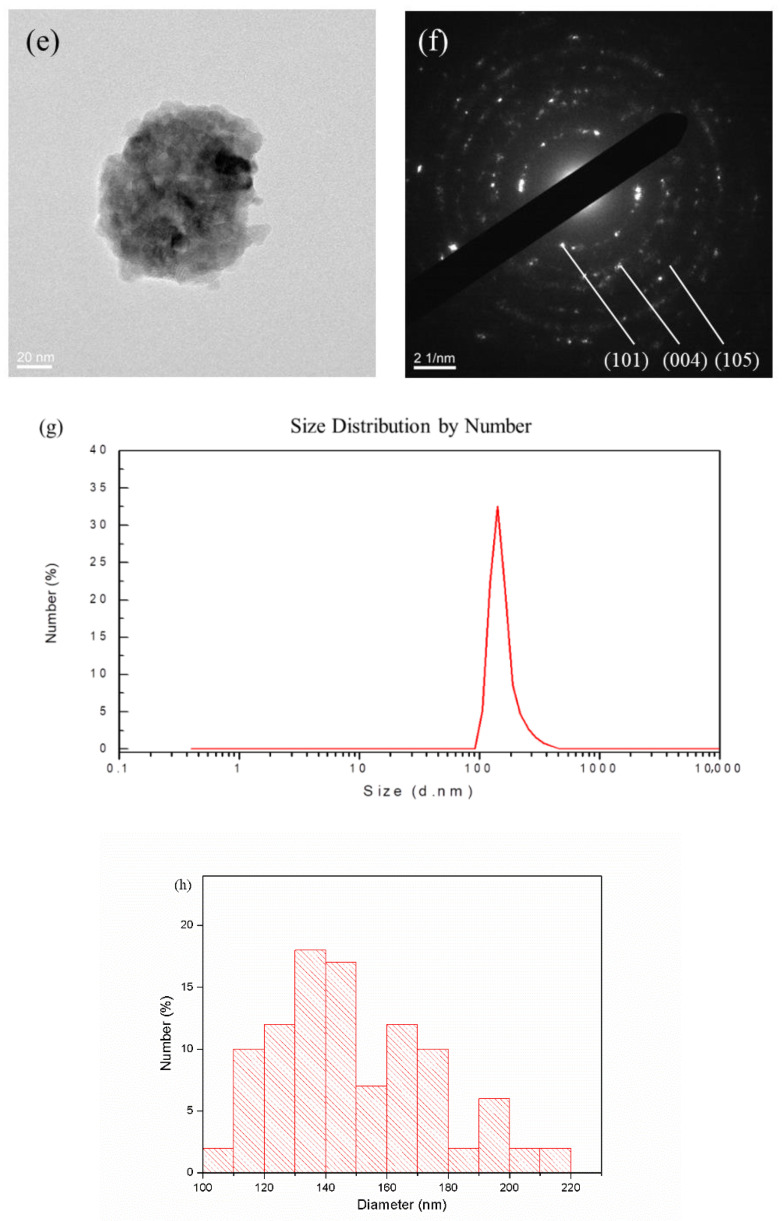
(**a**) XRD pattern of C-doped TiO_2_, (**b**) low-magnification SEM images of C-doped TiO_2_, (**c**) high-magnification SEM images of C-doped TiO_2_, (**d**) EDS of C-doped TiO_2_, (**e**) TEM images of C-doped TiO_2_, (**f**) Diffraction pattern of C-doped TiO_2_, (**g**) Size distribution of C-doped TiO_2_ and (**h**) SEM particle size distribution of C-doped TiO_2_.

**Figure 3:**
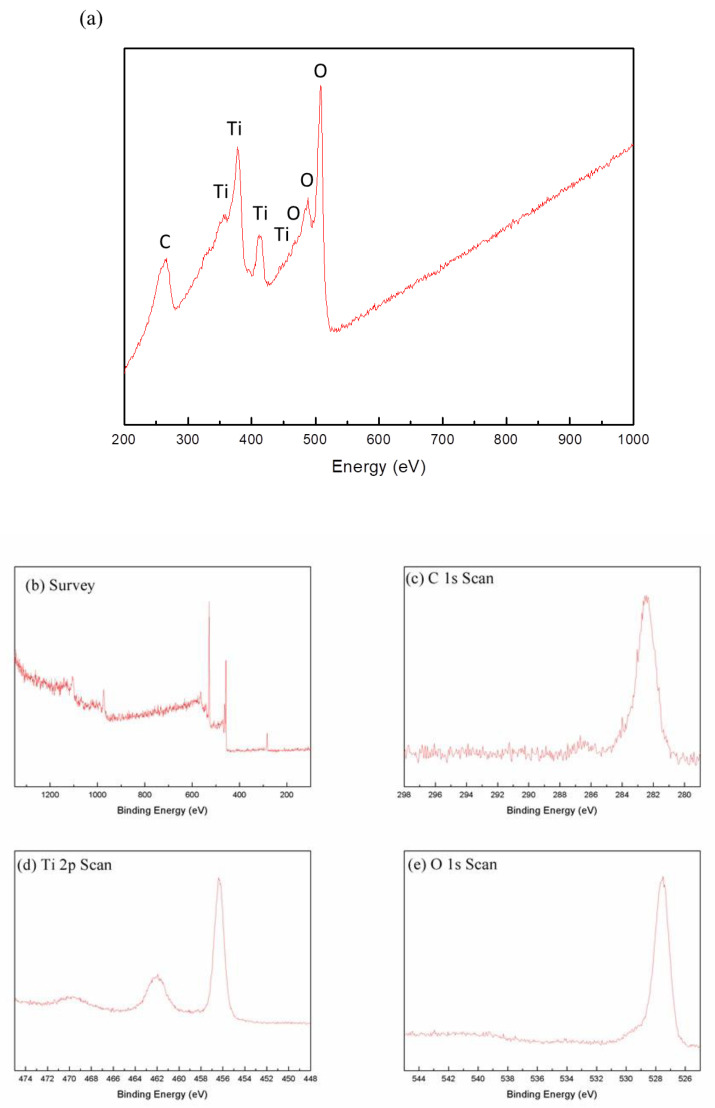
(**a**) AES spectra of C-doped TiO_2_, (**b**) XPS spectra of C-doped TiO_2_, (**c**) C 1s scan, (**d**) Ti 2p scan and (**e**) O 1s scan.

The XRD pattern of the synthesized C-doped TiO_2_ is shown in Figure 2a. It is indicated that the 2θ at 25.2°, 38.3°, 48°, 54.3° and 55.1° corresponded to the crystal form of (101), (004), (200), (105) and (211), which was completely matched with the standard pattern of the anatase structure of TiO_2_ (JCPDS Card No. 21-1272). The surface morphology of C-doped TiO_2_ was characterized by SEM as shown in Figure 2b,c. Figure 2d shows the chemical composition of the material analyzed by EDS, Ti, O and C elements with an atomic percent (At%) of 62.8, 32.8 and 4.4, respectively. The particles were spherical with a rough surface. The structure and diffraction pattern of the material were examined by TEM as shown in Figure 2e,f, respectively. C-doped TiO_2_ formed in the aggregation of nanograins, and its particle size was in the range of 100 to 200 nm. The selected area diffraction pattern (SADP) of C-doped TiO_2_ showed a ring pattern with the concentric rings from interior to outside, representing the crystal planes of (101), (004) and (105) which corresponded to the anatase phase. The particle size was precisely measured by Zetasizer as shown in Figure 2g. The precise particle size was 156.9 nm with a polydispersity index (PDI) of 0.137, which was in agreement with the analysis of SEM particle size (148.9 ± 26.3 nm) as shown in Figure 2h. Figure 3a shows the detection of chemical composition of the material by AES. In addition to the peaks related to Ti and O, C was detected at the energy of 263 eV. Figure 3b shows the XPS spectra of C-doped TiO_2_ was at a binding energy of 282, 462 and 562 eV, representing O 1s, Ti 2p and C 1s, respectively. Figure 3c shows the XPS spectra of the C 1s scan: the peak of TiC was detected at binding energy around 282 eV [29]. Figure 3d shows the XPS spectra of the Ti 2p scan, the peak of Ti^4+^ was detected at a binding energy of 462 (Ti 2p_1/2_) and 456 eV (Ti 2p_3/2_). Figure 3e shows the XPS spectra of the O 1s scan: the peak of (Ti^4+^/Ti^3+^)-O was detected at a binding energy of 527 eV.

**Figure 4:**
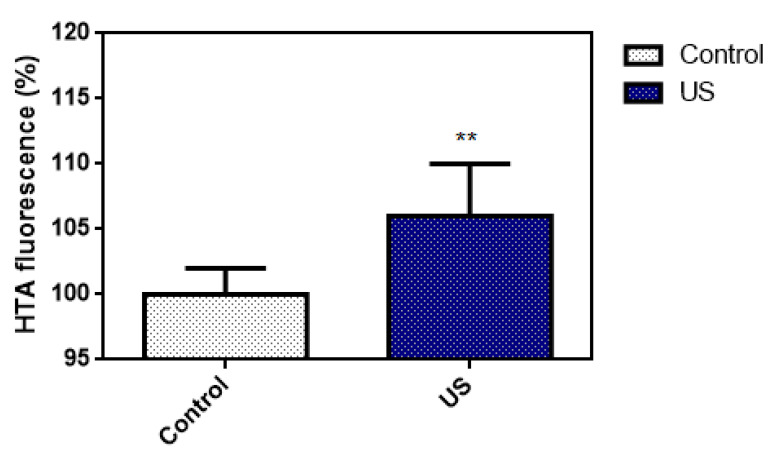
Oxidation of terephthalic acid (TA) to 2-hydroxyterephthalic acid (HTA) in the presence of hydroxyl radicals induced by inertial cavitation (one-way ANOVA, mean ± SD, *n* = 6, **: *p* < 0.01).

The 2-hydroxyterephthalic acid (HTA) fluorescence emission of the TA solution with or without US irradiation is shown in Figure 4. TA is a non-fluorescent compound and may further react with hydroxyl radicals to produce a highly fluorescent HTA. The fluorescence was 106 ± 4% (US group) and 100 ± 2% (control group), respectively. The fluorescence emission of the control group was 100% as the baseline. Each experiment was repeated 6 times. Based on the result, we believe that the US parameter in this study is able to induce inertial cavitation.

**Figure 5:**
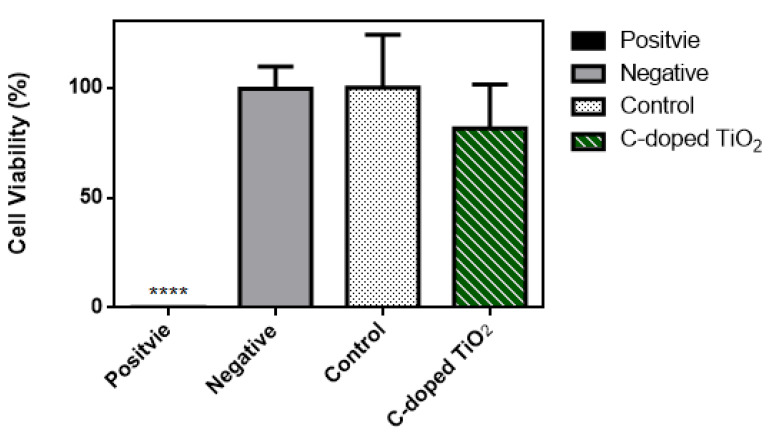
WST-1 test for the biocompatibility of C-doped TiO_2_ (one-way ANOVA, mean ± SD, *n* = 5, ****: *p* < 0.0001).

The biocompatibility test of C-doped TiO_2_ on cell viability by the WST-1 test is shown in Figure 5. After treatment with C-doped TiO_2_, the cell viability was 0.25 ± 0.24% (positive control group), 99.54 ± 10% (negative control group), 100 ± 24% (control group) and 81.49 ± 20% (C-doped TiO_2_ group), respectively. The cell viability of the control group was 100% as the baseline. Each experiment was repeated 5 times. The biocompatibility test followed by the regulations of ISO 10,993 indicated that there would be no potential toxicity when the cell viability of the experiment group was over 75%. Based on the WST-1 test, C-doped TiO_2_ showed no potential toxicity to normal cells.

**Figure 6:**
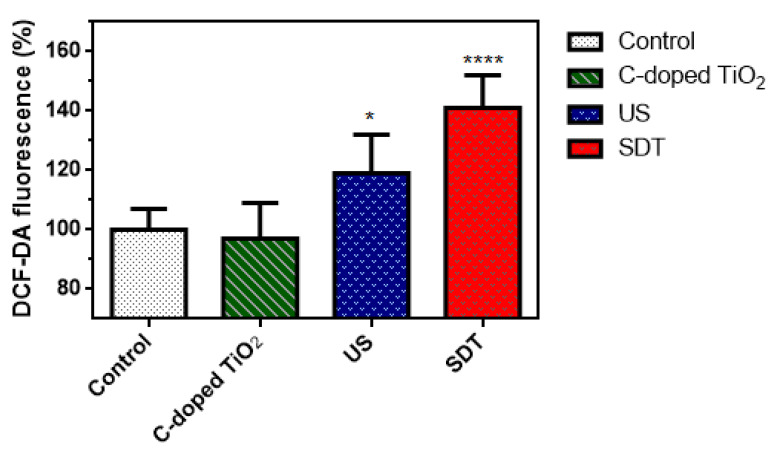
DCFDA test for relative ROS concentration induced by C-doped TiO_2_ activated by US (one-way ANOVA, mean ± SD, *n* = 6, *: *p* < 0.05, ****: *p* < 0.0001).

ROS generation of C-doped TiO_2_ under US irradiation by the DCFDA test is shown in Figure 6. DCFDA was able to permeate into 4T1 cells and be deacetylated by cellular esterases to a non-fluorescent DCFH. DCFH was then oxidized to a highly fluorescent 2′, 7′–dichlorofluorescein (DCF) in the presence of ROS. After the treatments, the fluorescence was 100 ± 7% (control group), 97 ± 12% (C-doped TiO_2_ group), 119 ± 13% (US group) and 141 ± 11% (SDT group), respectively. The fluorescence of the control group was 100% as the baseline. Each experiment was repeated 6 times.

**Figure 7:**
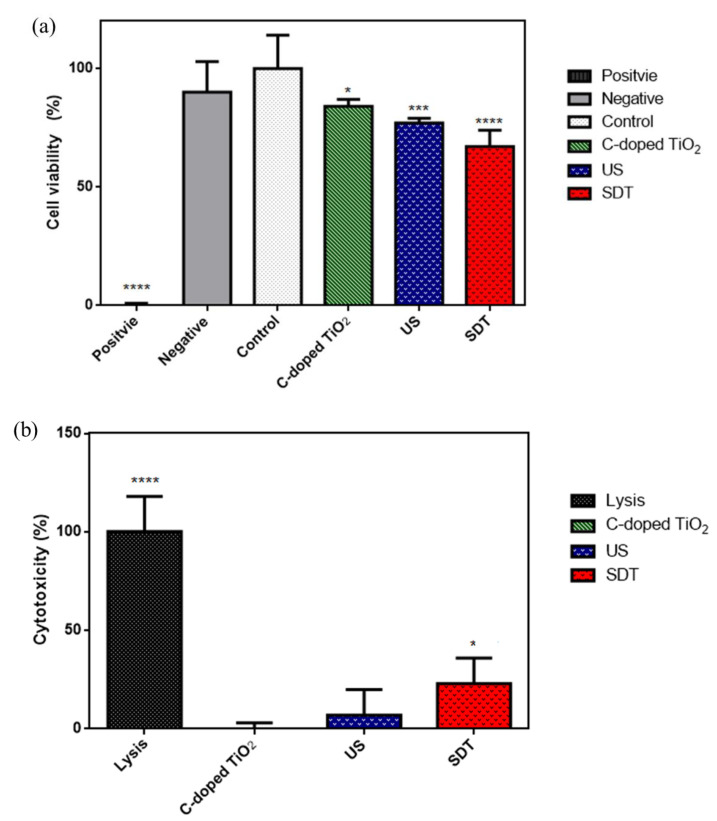
(**a**) Cell viability induced by C-doped TiO_2_ activated by US evaluated by WST-1 assay (one-way ANOVA, mean ± SD, *n* = 6), (**b**) cytotoxicity induced by C-doped TiO_2_ activated by US evaluated by LDH assay (one-way ANOVA, mean ± SD, *n* = 6, *: *p* < 0.05, ***: *p* < 0.001, ****: *p* < 0.0001).

The effect of C-doped TiO_2_ in SDT on cell viability and cytotoxicity of 4T1 cells was examined by WST-1 and LDH tests. The results of live/dead assay are shown in Figure S1 to evaluate cell viability in C-doped TiO_2_ during ultrasound irradiation. After the treatments, the cell viability was 0 ± 1% (positive control group), 90 ± 13% (negative control group), 100 ± 14% (control group), 84 ± 3% (C-doped TiO_2_ group), 77 ± 2% (US group) and 67 ± 7% (SDT group), respectively, as shown in Figure 7a. The cell viability of the control group was 100% as the baseline. Each experiment was repeated 6 times. After the treatments, the cytotoxicity was 100 ± 18% (total lysis group), 0 ± 3% (C-doped TiO_2_ group), 7 ± 13% (US group) and 23 ± 13% (SDT group), respectively, as shown in Figure 7b. The cytotoxicity of the control group was 0% as the baseline.

**Figure 8:**
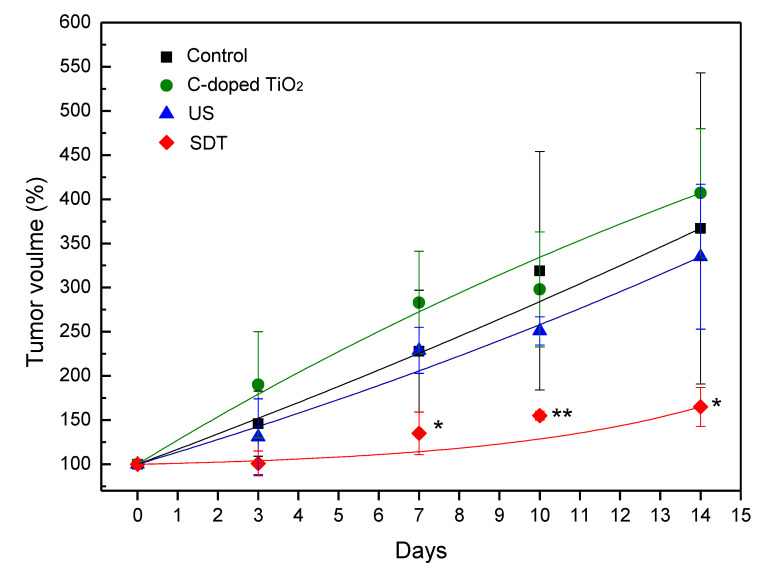
The variation of relative tumor volume in BALB/c nude mice induced by C-doped TiO_2_ activated by US (one-way ANOVA, mean ± SD, *n* = 5, *: *p* < 0.05, **: *p* < 0.01).

**Figure 9:**
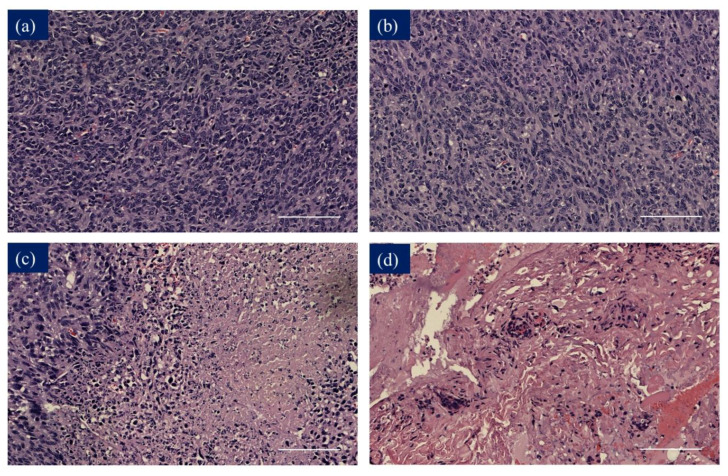
H&E-stained images of tumor tissue. (**a**) Control group, (**b**) C-doped TiO_2_ group, (**c**) US group and (**d**) sonodynamic therapy (SDT) group.

The antitumor efficacy of C-doped TiO_2_ under ultrasound irradiation was evaluated using BALB/c nude mice subcutaneously injected with 4T1 cells, where C-doped TiO_2_ particles were delivered into mice during the entire experiment and then treated with ultrasound irradiation twice—on day 0 and day 7 (Figures S2 and S3). The variation of relative tumor volume is shown in Figure 8, which was used to evaluate the antitumor effect of C-doped TiO_2_ in SDT for 4T1 cells. At 14 days, the relative tumor volume was 367 ± 176% (control group), 407 ± 73% (C-doped TiO_2_ group), 335 ± 82% (US group) and 165 ± 22% (SDT group), respectively. The H&E-stained images of organs are shown in Figure S4. The result showed that the C-doped TiO_2_ group and US group could not suppress the growth of the tumor. On the other hand, the SDT group could retard the tumor growth significantly (*p* < 0.05). The histologic staining of the tumor site is shown in Figure 9. H&E-stained tumor tissue images confirmed that SDT enhanced the ability to cause the death of the 4T1 cells compared to the other groups.

## 4. Discussion

TiO_2_ is one of most representative material studied in inorganic sonosensitizers. Nonetheless, the low quantum yield of ROS limits the effectiveness of TiO_2_ as a sonosensitizer by rapid recombination of the free electron and electron hole. The addition of noble metals, such as Pt and Au, have been reported to retard carrier combination [30,31]. Pt-doped or Au-doped TiO_2_ has been confirmed to show therapeutic efficacy and suppress the growth of tumors significantly. However, the price of novel metals would increase the cost of material preparation dramatically. Carbon shows highly promising dopant to narrow the bandgap, reaching similar therapeutic efficacy to a novel metal-based system in a more economical way. The comparative table is listed in Table 1.

In Figure 2a, the crystal structure of synthesized C-doped TiO_2_ was matched with the standard pattern of the anatase structure without significant structure change when carbon atoms played the substitutional foreign atoms, replacing some of the oxygen atoms. The results corresponded with the previous study [35]. In Figure 3c, the peak at around 282 eV was ascribed to Ti-C bonds. Thus, the Kröger–Vink notation of possible substitution of O^2−^ by C was proposed and shown in Equation (2). The creation of oxygen vacancies may form the impurity band that contributes a band tailing effect, thus diminishing the bandgap [36,37]. In the previous study [38], comparing the typical Ti-O binding energy, the clear blue-shift was shown, which indicated the presence of oxygen vacancy after the reduction.

(2) $$\mathrm{TiC}\;\overset{{TiO}_{2}}{\to }{\mathrm{Ti}}_{\mathrm{Ti}}^{\times }\;+\;{Co}^{\prime \prime }\;+\;{\mathrm{Vo}}^{••}$$

In Figure 2c,g, the particle size of C-doped TiO_2_ was 156.9 nm which made the nanoparticles possible to accumulate in tumor tissue without entering the normal tissue [39].

Mechanical index (MI) was one of the indicators for evaluating the occurrence of the inertial cavitation. MI was calculated using Equation (3), where P^-^ is the peak-negative acoustic pressure and *fc* is the center frequency of the ultrasound transducer. It was shown that 0.3 MPa of the peak-negative acoustic pressure was the least pressure to induce the inertial cavitation in aqueous solution [40]. In Figure 4, under US irradiation, the production of HTA increased significantly compared to the control group. In other words, US irradiation is able to elevate the OH generation in TA solution. Hence, the US parameter in this study was confirmed to be effective to induce the inertial cavitation.

(3) $$\mathrm{MI}\;=\;\frac{P}{\sqrt{f_{c}}}$$

**Figure 10:**
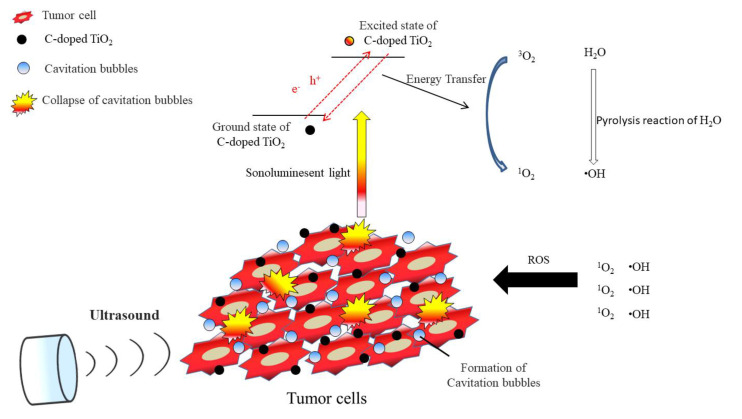
The possible pathway of 4T1 tumor cell damage induced by SDT.

In Figure 6, compared to the control group, 4T1 cells treated with C-doped TiO_2_ did not increase the level of ROS; 4T1 cells treated with US could be effective to increase the level of ROS, ascribed to the pyrolysis reaction of the water to produce hydroxyl radicals; 4T1 cells treated with C-doped TiO_2_ plus US could increase the most significant level of ROS than the other groups, ascribed to the highly reactive singlet oxygen and hydroxyl radicals. This result corroborated that, under US irradiation, ROS generation could be increased in the presence of C-doped TiO_2_. However, the amount of extracellular generation of ROS may affect the cell toxicity of tumor cells. Figure 7 shows that SDT induced higher toxicity in 4T1 cells than the other three groups. The results show that only US treatment seemed to cause insufficient ability to damage the 4T1 cells with the production of hydroxyl radicals. Nonetheless, US treatment in the presence of C-doped TiO_2_ seemed to significantly suppress the proliferation of 4T1 cells. The cellular uptake of C-doped TiO_2_ to 4T1 cells was supposed to occur via endocytosis or pinocytosis [41]. The possible pathway of cell damage induced by SDT may be speculated as: Under US irradiation, C-doped TiO_2_ was activated by the sonoluminescent light, induced by inertial cavitation, to an excited state. Carbon was doped in the anatase TiO_2_ to form a new valence band, thus narrowing the bandgap. The addition of carbon may give TiO_2_ an excess of conducting electrons or holes, which is important for lowering the bandgap [23,24]. The unstableness of the excited state may cause decay back to the ground state, leading to energy release. The released energy may transfer to oxygen to generate the highly reactive singlet oxygen and water to generate hydroxyl radicals (Figure 10). With the increasing concentration of singlet oxygen and hydroxyl radicals, 4T1 cells would be gradually damaged and lead to cell senescence and death due to oxidative damaging effects [11,42,43]. Due to the enhanced permeability and retention (EPR) effect, C-doped TiO_2_ preferentially accumulates in tumor cells eliciting efficient ROS generation [44] and further increasing the effectiveness of the SDT treatment. It was also revealed that the suppression of the tumor is due to the elevated level of ROS which results in both direct tumor cell death and blood vessel stasis. ROS can induce blood stasis via platelet aggregation or vessel constriction by destroying the endothelial layer [45]. Furthermore, SDT may elevate the level of inflammatory-associated cytokine (including TNF-alpha, IL-6, IL-1) production which is known to stimulate the maturation and function of granulocytes and macrophages [46].

To further investigate the cell death of 4T1 cells induce by SDT, in-vivo study was implemented in this study. Figure 8 shows that SDT could be more effective in suppressing the growth of the 4T1 tumor with a significant difference between SDT and the other groups on day 7. The relative tumor volume of SDT ended with almost half compared to the other groups at the 14th day of the treatment. H&E-stained tumor tissue is shown in Figure 9. In the tumors treated with 1× PBS or C-doped TiO_2_ individually, integral tumor cellularity with spindle shape nuclei was visible. In the tumors treated with US, attributed to the inertial cavitation effect of US, a small area of nuclear fragmentation with little necrosis was visible. However, in the tumors treated with SDT, with the synergistic effect of hydroxyl radicals and singlet oxygen, a huge necrotic area with shrinkage and fragments of nuclei was seen. These results corroborated that SDT could inhibit the proliferation of 4T1 tumor cells.

## 5. Conclusions

The availability of C-doped TiO_2_ nanoparticles as sonosensitizers activated by low-intensity ultrasound to generate ROS for cancer treatment was investigated in this study. Spherical C-doped TiO_2_ nanoparticles with an average particle size of around 157 nm were synthesized by the sol-gel process. From the WST-1 test, C-doped TiO_2_ showed no potential toxicity to normal cells. From in vitro study, it showed more extracellular generation of ROS in 4T1 breast cancer cells under SDT (C-doped TiO_2_/US treatment). Moreover, the effect of C-doped TiO_2_ in SDT could reduce the cell viability and increase the cytotoxicity of 4T1 cells. From in vivo study, the antitumor effect of C-doped TiO_2_ in SDT may inhibit the proliferation of the 4T1 tumor and lead to a huge necrotic area with shrinkage and fragments of nuclei in the tumor site. We believe that C-doped TiO_2_ nanoparticles could be viewed as sonosensitizers in SDT activated by low-intensity ultrasound for ROS generation as an effective strategy for alternative cancer treatments in the future.

## Figures and Tables

**Table 1:** The sonosensitizers and the matched ultrasound parameters.

Tumor/Source	Host	Sonosensitizers	Ultrasound Parameter			Reference
			MHz (f)	W/cm^2^ (I)	Duration (s)	
Heptaic/human	mouse	TiO_2_	0.5/1.0	0.8/0.4	60	[32]
Skin/mouse	mouse	TiO_2_	1	1.0	120	[33]
Breast/human	mouse	TiO_2_	1	0.1	30	[34]
Lung/mouse	mouse	Au-doped TiO_2_	1.5	30	30	[31]
Breast/human	mouse	Pt-doped TiO_2_	1	1.5	300	[30]

## Supplementary Materials

The following are available online at https://www.mdpi.com/2076-3921/9/9/880/s1, Figure S1: Live/Dead assay; Figure S2: survival rate of BALB/c nude mice; Figure S3: average weight of BALB/c nude mice; Figure S4: H&E-stained images of organs.
