# Supplementary material for: Using C-doped TiO2 Nanoparticles as a Novel Sonosensitizer for Cancer Treatment
Source: Antioxidants (Basel). 2020 Sep 17;9(9):880. doi: 10.3390/antiox9090880 (PMC7554704; doi:10.3390/antiox9090880)
Supplement: Supplementary file 1 [file antioxidants-09-00880-s001.pdf]

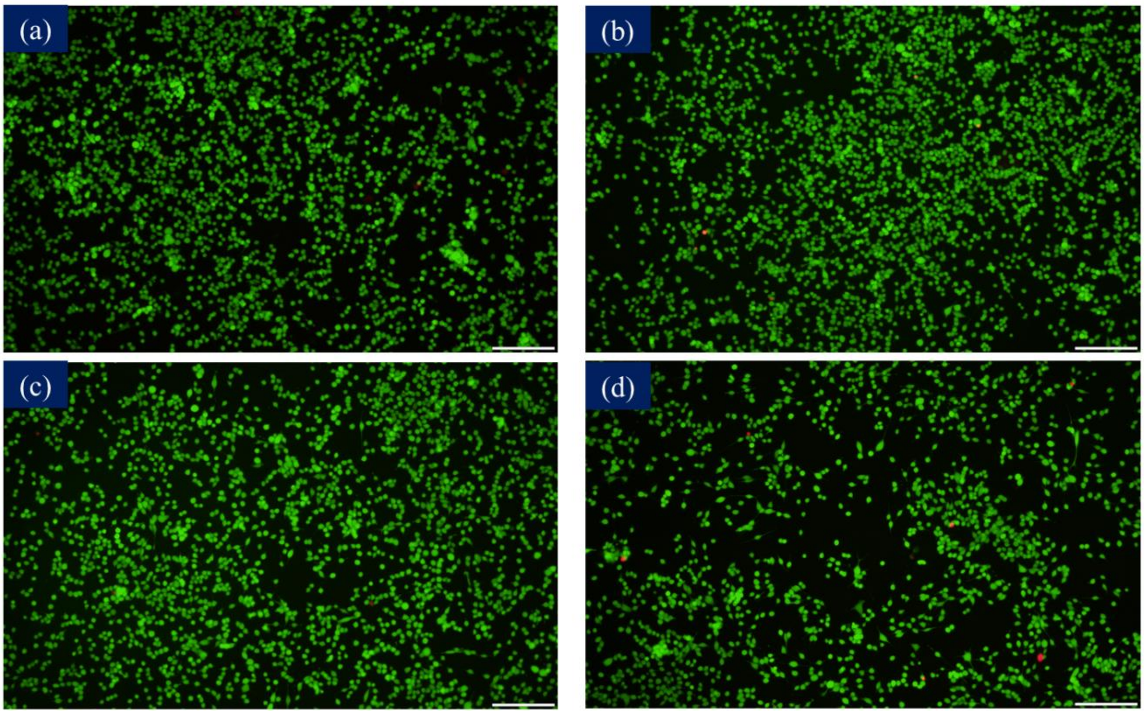


Fig. S1 Live/Dead assay. (a) control group (b) C-doped $TiO_2$ group (c) US group (d) SDT group

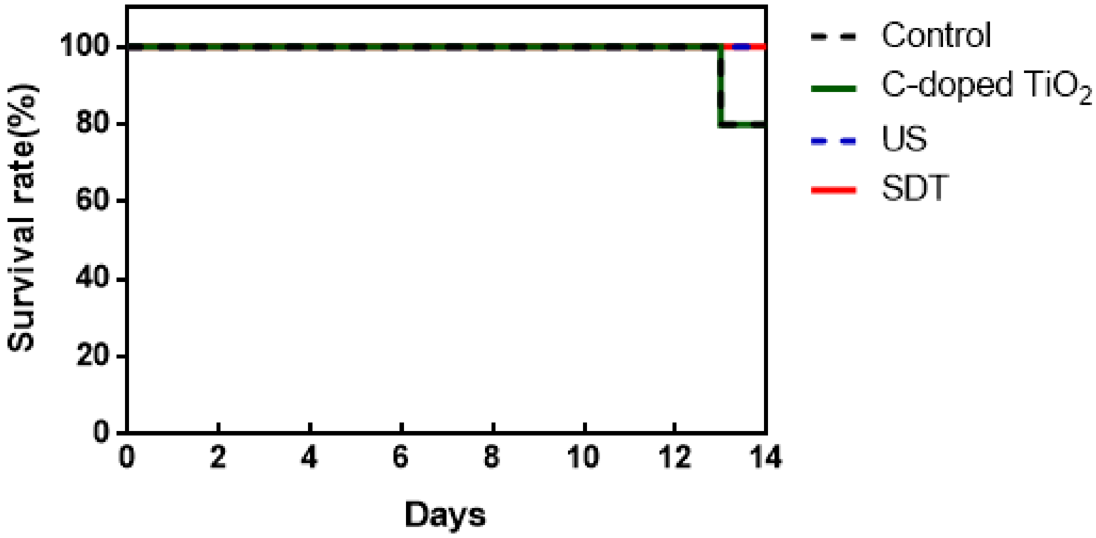


Fig. S2 Survival rate of BALB/c nude mice (n = 5)

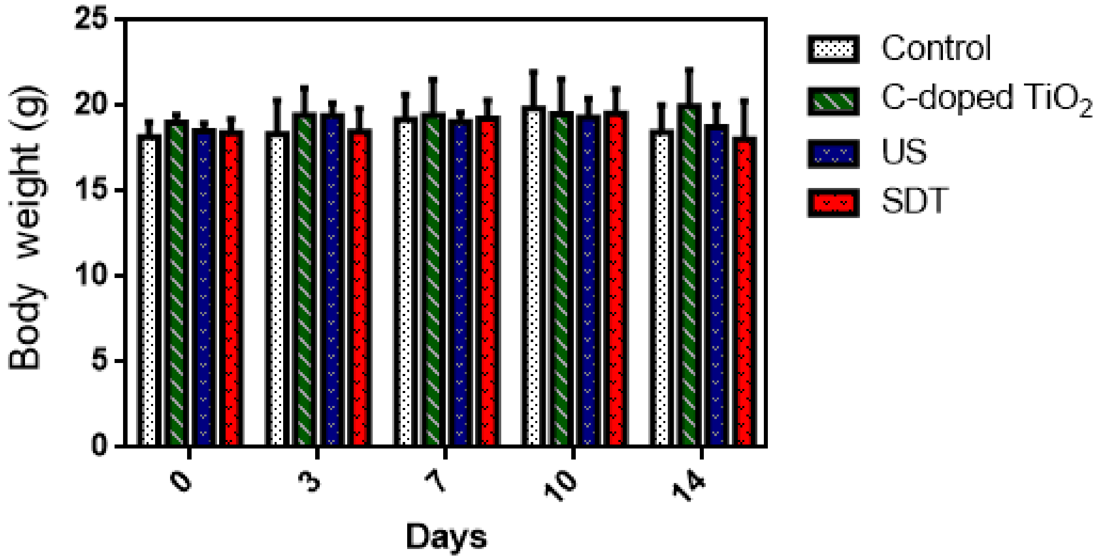


Fig. S3 Average weight of BALB/c nude mice (one-way ANOVA, mean ± SD, n = 5)

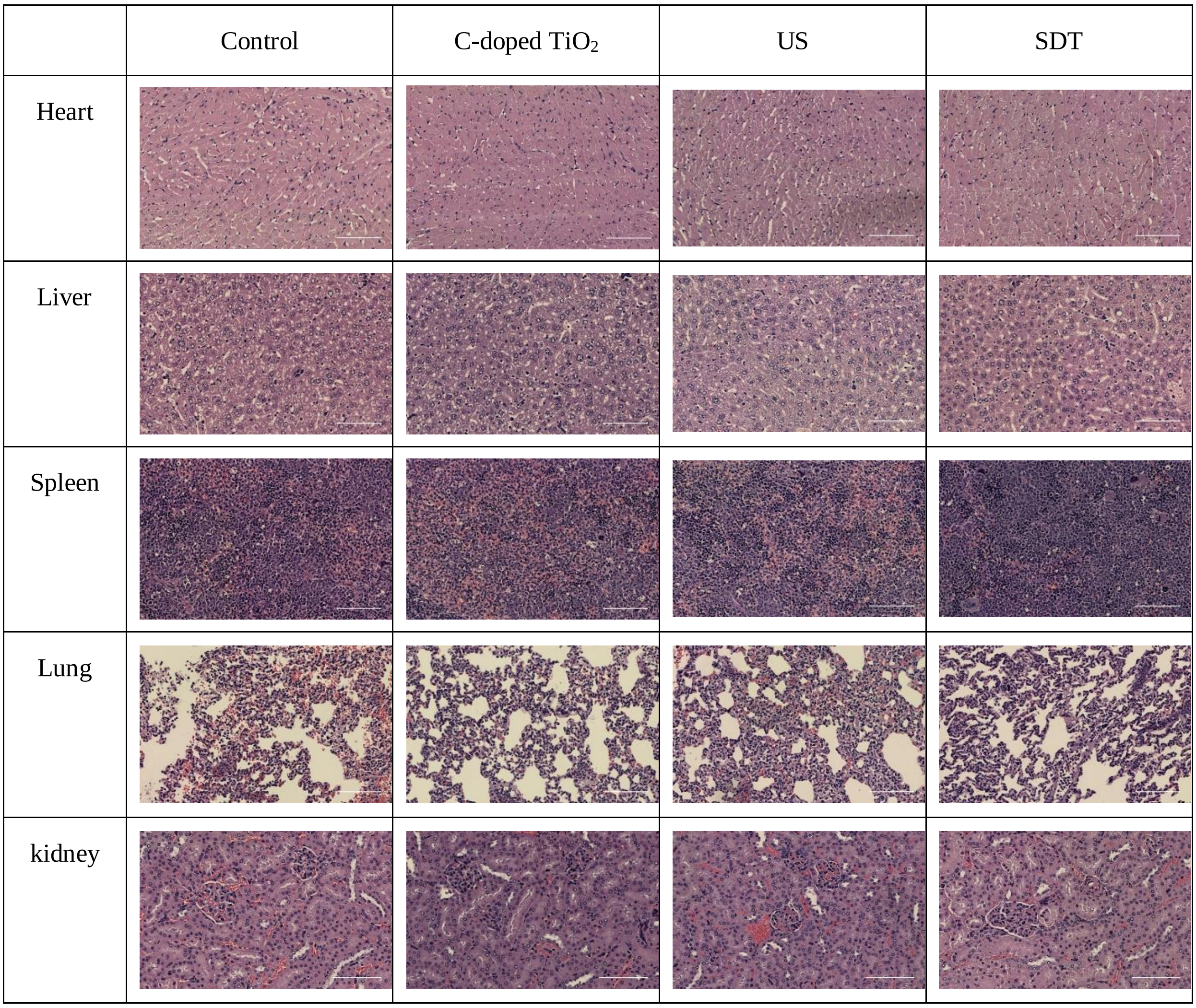


Fig. S4 H&E-stained images of organs.
